# A systematic review of reviews on the prevalence of anxiety disorders in adult populations

**DOI:** 10.1002/brb3.497

**Published:** 2016-06-05

**Authors:** Olivia Remes, Carol Brayne, Rianne van der Linde, Louise Lafortune

**Affiliations:** ^1^ Department of Public Health and Primary CareUniversity of CambridgeCambridgeCB1 8RNUK; ^2^ London Borough of Hammersmith and FulhamWestminster City CouncilLondonSW1E 6QPUK

**Keywords:** Anxiety, anxiety disorders, demographics, epidemiology, international, mental disorders, prevalence

## Abstract

**Background:**

A fragmented research field exists on the prevalence of anxiety disorders. Here, we present the results of a systematic review of reviews on this topic. We included the highest quality studies to inform practice and policy on this issue.

**Method:**

Using PRISMA methodology, extensive electronic and manual citation searches were performed to identify relevant reviews. Screening, data extraction, and quality assessment were undertaken by two reviewers. Inclusion criteria consisted of systematic reviews or meta‐analyses on the prevalence of anxiety disorders that fulfilled at least half of the AMSTAR quality criteria.

**Results:**

We identified a total of 48 reviews and described the prevalence of anxiety across population subgroups and settings, as reported by these studies. Despite the high heterogeneity of prevalence estimates across primary studies, there was emerging and compelling evidence of substantial prevalence of anxiety disorders generally (3.8–25%), and particularly in women (5.2–8.7%); young adults (2.5–9.1%); people with chronic diseases (1.4–70%); and individuals from Euro/Anglo cultures (3.8–10.4%) versus individuals from Indo/Asian (2.8%), African (4.4%), Central/Eastern European (3.2%), North African/Middle Eastern (4.9%), and Ibero/Latin cultures (6.2%).

**Conclusions:**

The prevalence of anxiety disorders is high in population subgroups across the globe. Recent research has expanded its focus to Asian countries, an increasingly greater number of physical and psychiatric conditions, and traumatic events associated with anxiety. Further research on illness trajectories and anxiety levels pre‐ and post‐treatment is needed. Few studies have been conducted in developing and under‐developed parts of the world and have little representation in the global literature.

## Introduction

Anxiety disorders – defined by excess worry, hyperarousal, and fear that is counterproductive and debilitating – are some of the most common psychiatric conditions in the Western world (Simpson et al. 2010). The prevalence of anxiety disorders in the United States is estimated to be 18% (Kessler et al. 2005), and their annual cost is reported to be $42.3 billion (Greenberg et al. 1999). In the European Union (EU), over 60 million people are affected by anxiety disorders in a given year, making them the most prevalent psychiatric conditions in the EU (Wittchen et al. 2011). The Global Burden of Disease (GBD) study estimated that anxiety disorders contributed to 26.8 million disability adjusted life years in 2010. (Whiteford et al. 2013). While a number of reviews have focused on the burden of depression and its economic, social, and health care policy implications, substantially fewer have assessed anxiety.

The past decade has seen increased research interest into anxiety disorders, in large part because of a greater recognition of their burden and the implications associated with untreated illness. Clinical reviews have shown that the presence of an anxiety disorder is a risk factor for the development of other anxiety and mood disorders and substance abuse. In clinical and population‐based studies, the development of comorbidities makes the treatment of primary and secondary disorders difficult, contributes to low remission rates, poor prognosis and risk of suicide (Nutt and Ballenger 2003; Simpson et al. 2010). Untreated anxiety has been associated with significant personal and societal costs, related to frequent primary and acute care visits, decreased work productivity, unemployment, and impaired social relationships (Simpson et al. 2010).

A number of primary studies on the prevalence of anxiety have been undertaken, but the variability in findings has made generalizability to the wider population difficult. This variability mainly results from differences in study setting (i.e., culture; clinical vs. population‐based), age and sex composition of samples, length of follow‐up, methods of anxiety assessment, and caseness criteria (i.e., types and number of disorders examined). Systematic reviews on the prevalence of these conditions in highly select, homogeneous population subgroups have been undertaken, but the selective citation of such estimates presents a distorted view of the overall burden of anxiety and limits generalizability.

The aim of this systematic review of reviews was to provide a comprehensive synthesis and description of the prevalence of anxiety disorders in the general population, as well as in clinical outpatient and inpatient groups affected by a range of chronic physical diseases and psychiatric disorders, as reported by individual reviews. Individuals recruited from the community can have different risk factor profiles than those sampled from clinical settings, potentially giving rise to different rates of mental health problems amongst these groups (Nutt and Ballenger 2003; Simpson et al. 2010). As a result, the burden needs to be assessed across different settings and segments of the population. To provide insight into the demographic groups that are most affected, we reported on estimates for men and women and different age groups, if this information was available. Since a number of studies (Walters et al. 2004; Skapinakis et al. 2005; Simpson et al. 2010) have identified the need to better understand the geographical variation of mental health problems, we included reviews that captured studies conducted across the globe at national and subnational levels. To provide insight into the chronicity of anxiety disorders, we provided period (i.e., 12‐month) and lifetime prevalence estimates. If the duration criterion was not clearly stated or the “point” or “current” prevalence was indicated, we simply referred to these estimates as “prevalence”.

Findings from this systematic review will shed light on the groups that are most affected by anxiety disorders, and can be used to inform targeted screening and treatment efforts. This will be important in the planning of health services and the development of evidence‐based policy. Finally, results from this review can be used to identify areas where further research is needed.

This is the first study to provide a comprehensive synthesis of the disparate findings from systematic reviews undertaken on the burden of anxiety across the globe and using a systematic approach.

## Methods

### Search strategy

We defined a systematic review in accordance with the Cochrane Collaboration and the Preferred Reporting Items for Systematic Reviews and Meta‐Analyses (PRISMA) Statement (Moher et al. 2009). (Appendix 1) We included high‐quality reviews that reported the prevalence of anxiety disorders in the general population or clinic‐based settings. We searched for reviews on young, middle‐aged, and older adults with risk behaviors (i.e., drug abuse), chronic or infectious diseases, psychiatric conditions, who are vulnerable, and living in countries across the globe. Reviews on the treatment of anxiety were not included, as we consider this to be a separate review topic that would merit an in‐depth analysis.

To identify reviews meeting the inclusion criteria, we searched Medline (inception‐May, 2015), PsycInfo (1987‐May, 2015), and Embase (inception‐May, 2015) using combinations of keywords relating to anxiety and prevalence (Appendix 2). Reference lists were hand‐searched for additional reviews. Titles and abstracts of non‐English language articles were translated to assess relevance. We excluded unpublished data. The review protocol is registered on PROSPERO (Remes et al. 2014).

### Inclusion criteria

We searched for reviews that reported the lifetime, period, or point prevalence (or simply “prevalence”) of generalized anxiety disorder (GAD), obsessive‐compulsive disorder (OCD), social anxiety disorder (SAD) or social phobia, agoraphobia, panic disorder (PD) with or without agoraphobia, and simple or specific phobia, and anxiety not otherwise specified (NOS). Studies that reported the prevalence of aggregated anxiety disorders, subthreshold disorders, or anxiety symptoms were also included. Reviews were included regardless of the sampling framework used in primary studies.

Reviews were included regardless of the method of anxiety assessment. Specifically, reviews capturing primary studies on threshold and subthreshold disorders that were assessed through fully, semi‐, or unstructured interviews administered by clinicians or trained professionals, symptom checklists, clinician diagnoses, and self‐report were accepted. Interviews or self‐reported questionnaires that mapped to standard classificatory systems, such as the Diagnostic and Statistical Manual of Mental Disorders (DSM) (American Psychiatric Association, 2010) or the International Classification of Diseases (ICD) (World Health Organization, 2016), were also included.

OR and LL screened titles and abstracts, and disagreements were resolved through discussion. Dissertations, case reports, letters, and commentaries were excluded. Full‐text articles were retrieved for further assessment by OR.

### Quality assessment

Quality assessment of the reviews meeting the inclusion criteria was undertaken by OR and RvdL. If reviews met at least five of the criteria stipulated by AMSTAR (Shea et al. 2009), a validated measurement tool for assessing the quality of systematic reviews, they were included. For example, some of the AMSTAR quality criteria assess whether an “*a priori*” design was established, whether there was duplicate study selection and data extraction, if the literature search was comprehensive, whether the quality of primary studies was examined, etc.

### Data extraction and analysis

Data extraction was performed by OR and RvdL using the standardized form capturing device: the dates of publication and literature search; objectives; number of studies reviewed; prevalence of anxiety; sample characteristics; sample size range of primary studies; recommendations for future research, and limitations of primary studies and review. Disagreements were resolved through discussion.

Studies were grouped according to five common themes and prevalence was described in the context of: (1) addiction, (2) other mental and neurological disorders, (3) chronic physical diseases, (4) trauma, and (5) vulnerable population subgroups. If there were fewer than three reviews on a chronic physical disease, it was grouped under: “other chronic physical diseases” or “other chronic physical diseases in end‐stage”. Vulnerable population subgroups refer to individuals at high risk for poor health, who may experience stigma, marginalization, or health service access barriers.

We did not perform a meta‐analysis because of the heterogeneity in study methodology. Quantitative measurement of heterogeneity was not undertaken. Finally, a meta‐analysis of primary studies included in 48 systematic reviews would not have been feasible. We described the prevalence of individual and aggregate anxiety disorders, subthreshold disorders, or symptoms of anxiety, as reported by the systematic reviews. If reviews provided clear prevalence estimates for men and women and different age groups, we also included this information.

## Results

**Figure 1:**
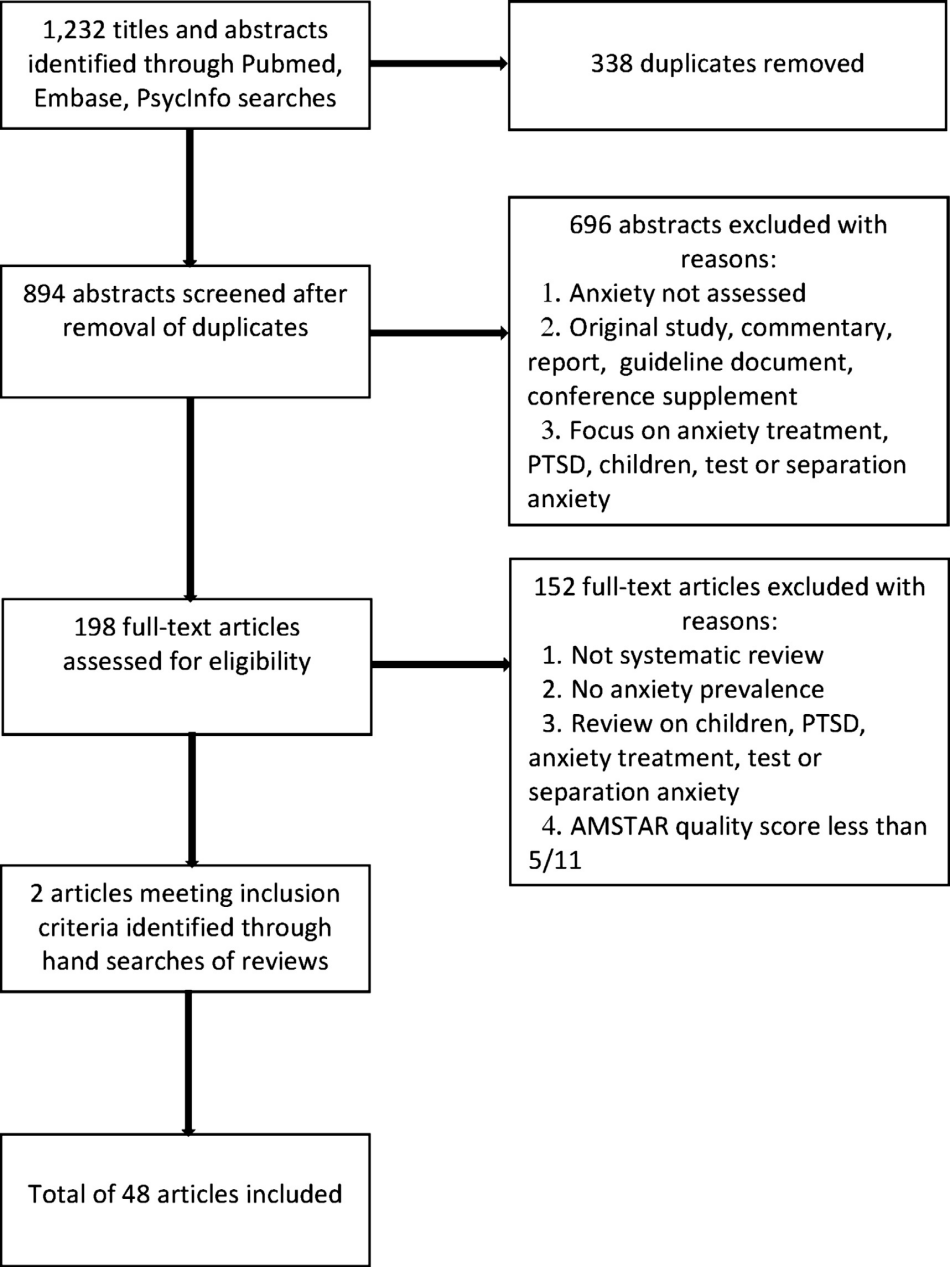
Flowchart of main search strategy and article selection for systematic review of reviews.

The search identified 1232 reviews on anxiety. After 338 duplicates were removed, titles and abstracts were screened, and the full text of 198 articles was retrieved. In total, 46 systematic reviews met the inclusion criteria (Fig. 1). Reference searches identified two additional reviews as relevant, yielding a total of 48 reviews in this systematic review (Appendix 3).

Of the 48 reviews, seven focused on the descriptive epidemiology of anxiety disorders, while five reviewed anxiety in relation to addiction. Four focused on mental and neurological disorders. A total of 19 reviews assessed anxiety in the context of chronic physical diseases: most of these focused on CVD (*n* = 6) and cancer (*n* = 7), followed by respiratory disease (*n* = 3) and diabetes (*n* = 3); the rest examined end‐stage physical disease (*n* = 4), and conditions that have been less commonly studied in the anxiety field (*n* = 4). Three reviews examined anxiety in the context of trauma, and ten focused on vulnerable population subgroups. Most of the reviews included international studies.

### The global distribution of anxiety disorders

Seven reviews focused on the descriptive epidemiology of anxiety disorders, presenting age‐, sex‐, and time trends. In one international review (Somers et al. 2006), the pooled one‐year and lifetime prevalence of total anxiety disorders was estimated to be 10.6% (95% CI: 7.5%, 14.3%) and 16.6% (95% CI: 12.7%, 21.1%), respectively. Given the health care policy and service planning implications of high estimates, a high‐quality meta‐analysis (Baxter et al. 2014) investigated whether the age‐standardized point prevalence of anxiety increased over the last decade. Studies on cultures across the globe were reviewed and findings showed that the prevalence in 1990 (3.8% [95% CI: 3.6%, 4.1%] was very similar to that in 2005 and 2010 (4.0% [95% CI: 3.7%, 4.2%]). A sharp rise in younger people over time was noted, but changing age and population structures were hypothesized to be the drivers of this. Prevalence was found to be lowest in East Asia (2.8% [95% CI: 2.2%, 3.4%]) and highest in North America (7.7% [95% CI: 6.8%, 8.8%]) and the North African/Middle Eastern region (7.7% [95% CI: 6.0%, 10%]) (Baxter et al. 2014). A less rigorous review (Somers et al. 2006) estimated the highest lifetime prevalence of anxiety disorders in Swiss and US populations (23–28.7%), and the lowest in studies on Korea (9.2%). In Pakistan (Mirza and Jenkins 2004), the prevalence of total anxiety ranged from 1.76% to 25%, while a meta‐analysis on Germany (Vehling et al. 2012) reported it to be 13.5% (95% CI: 7.1%, 24.3%).

Women are almost twice as likely to be affected as men (female:male ratio of 1.9:1), with sex differences persisting over time and across high and low resource settings (Somers et al. 2006; Baxter et al. 2013; Steel et al. 2014). Irrespective of culture, individuals under the age of 35 years are disproportionately affected by anxiety disorders (Baxter et al. 2013, 2014) with the exception of Pakistan, where midlife represents a period of high burden (Mirza and Jenkins 2004).

Globally, specific phobia (4.9% [95% CI: 3.4%, 6.8%] and GAD (6.2% (95% CI: 4.0%, 9.2%) appear to have the highest lifetime prevalence, and panic disorder the lowest (1.2% [95% CI: 95% CI: 0.7%, 1.9%]) (Somers et al. 2006). In Germany, however, specific phobia (5.2%, [95%CI: 3.3%, 8.2%]) and GAD (3.7%, [95% CI: 2.3%, 6.0%]) are reported to be the most prevalent anxiety disorders (Vehling et al. 2012). In addition to geographical variation, caseness criteria are an important consideration when comparing estimates. One review reported an almost twofold higher prevalence of subthreshold GAD when the duration criterion was relaxed from 3 to 1 month (3.6% vs. 6.1%). In this review, older age groups showed the lowest estimates of past‐year subthreshold GAD (3%) (Haller et al. 2014).

### Addiction

Five reviews focused on anxiety experienced in relation to addictive behaviors, including substance misuse, pathological gambling, and compulsive internet use. A global review on nonmedical prescription opioid use (NMPOU) reported the overall lifetime anxiety prevalence in patients at admissions or in treatment for substance abuse problems to range from 2% to 67% (Fatseas et al. 2010). While the prevalence of anxiety diagnoses is reportedly high at 29% (95% CI: 14%, 44%), that of subthreshold anxiety is higher still, with half of NMPOU populations enrolled in substance abuse treatment in North America reporting symptoms (50% [95% CI: 16%, 84%]) (Goldner et al. 2014). In contrast, general population samples of NMPOU in North America show a substantially lower prevalence of anxiety (16% [95% CI: 1%, 30%]) (Fischer et al. 2012). No significant age or sex‐effects were found in NMPOU groups enrolled in substance use treatment (Goldner et al. 2014).

Two other risk behaviors that have received attention in the addiction field include problem and pathological gambling, and more recently, internet addiction. When a global meta‐analysis assessed 11 community samples of pathological gamblers, the prevalence of anxiety disorders was reported to be 37.4% (Lorains et al. 2011). The prevalence of anxiety in the context of internet addiction is lower and comes mostly from studies conducted in Asian countries. A meta‐analysis found the prevalence of anxiety to be over two times higher in community samples of people with Internet addiction compared to control subjects (23.3% [95% CI: 14.8, 34.8%] vs 10.3% [95% CI: 5.0, 19.9%]), with those under the age of 39 being most affected (Ho et al. 2014).

### Other mental and neurological disorders

In Europe, approximately 13–28% of people with bipolar disorder recruited from clinical and community settings have comorbid anxiety, with GAD and panic disorder being frequently experienced by this population (Fajutrao et al. 2009). In the US and Italian samples with bipolar disorder (Amerio et al. 2014), OCD is also common. The prevalence of this anxiety disorder in those who are bipolar has been shown to range from 11.1% to 21% in population‐based studies, and 1.8% to 35.1% in clinical samples.

OCD is also highly comorbid with schizophrenia. A global review (Swets et al. 2014) estimated the prevalence of this disorder in people diagnosed with schizophrenia to be 12.3% (95% CI: 9.7%, 15.4%). The prevalence of obsessive compulsive symptoms (OCS) not meeting full caseness criteria was over twice that of OCD (30.7% [95% CI: 23%, 39.6%]). Lower anxiety prevalence was linked to sub‐Saharan African origin. Age and sex did not influence OCD or OCS rates (Swets et al. 2014). These estimates were mainly based on groups from clinical settings.

One of the highest prevalence figures of psychopathology was found by a review on multiple sclerosis (MS) (Marrie et al. 2015), which reported that almost 32% of people with MS have an anxiety disorder and over half experience symptoms. Some of the primary studies included in this review were based on participants recruited from the general population, suggesting that men and women with MS are at high risk for psychopathology. Health anxiety may be an important issue in this population subgroup, given that 26.4% of those with MS are affected. Study methodology made a significant contribution to the figures reported. Estimates of anxiety prevalence were substantially higher if they were derived through self‐reported questionnaires (25.5% [95% CI: 16.7%, 34.3%]) compared to administrative databases or medical records (15.4%, [95% CI: 0%, 39.0%]) (Marrie et al. 2015).

### Chronic physical diseases

#### Cardiovascular disease

Six reviews reported the prevalence of anxiety in the context of cardiovascular disease (CVD). Approximately a tenth of patients with cardiovascular disease and living in Western countries are affected by GAD (10.94% [95% CI: 7.8%, 14.0%] (Tully and Cosh 2013), with women showing higher anxiety levels than men (Clarke and Currie 2009). Anxiety symptom prevalence among patients with congestive heart failure is 2–49% (Janssen et al. 2008), and in end‐stage patients suffering from heart disease, it is 49% (Solano et al. 2006). Further, panic disorder is a common diagnosis in patients with coronary artery disease, with the prevalence ranging from 10% to 50% in this subgroup (Clarke and Currie 2009).

Individuals with noncardiac or nonspecific chest pain presenting to emergency departments, particularly women and those who are younger, appear to be disproportionately affected by anxiety. Compared to those with a determined cause of chest pain, anxiety prevalence was found to be higher in those with unknown etiology (21–53.5% of noncardiac chest pain patients have probable anxiety) (Webster et al. 2012).

A high‐quality, global meta‐analysis of population‐, hospital‐, and rehabilitation‐based studies found the prevalence of anxiety disorders in stroke patients to vary between 18% (95% CI: 8%, 29%) and 25% (95% CI: 21%, 28%) when measured by clinical interview and rating scales, respectively (Campbell Burton et al. 2013). Age and sex did not influence the probability of having anxiety after stroke in most of the included studies. GAD and phobic disorders were the commonest anxiety disorders post‐stroke.

#### Cancer

Seven reviews assessed anxiety among individuals diagnosed with or receiving treatment for cancer and in spouses of cancer patients. The prevalence of anxiety among cancer patients varies between 15% and 23%, with symptoms rising to 69–79% in the later stages of disease. There was no reported evidence with respect to age and sex (Solano et al. 2006; Clarke and Currie 2009).

A meta‐analysis (Yang et al. 2013) on working‐age and older adults living in Mainland China showed that the overall prevalence of anxiety in individuals with a cancer diagnosis was higher than that in noncancer controls (49.7% [95%CI: 20.0%, 89.1%] and 17.5%, respectively). Among German patients with breast cancer, the prevalence of anxiety was comparatively lower than in Chinese patients, ranging from 28.0% to 33.0% (Vehling et al. 2012).

Randomized controlled trials (RCT) and non‐RCT studies conducted across the globe showed that approximately a fourth to over half of individuals undergoing or who had undergone breast cancer treatment experienced anxiety (Lim et al. 2011). Lower levels of anxiety were observed in patients undergoing radiotherapy rather than chemotherapy. During chemotherapy, young age and high trait anxiety measured before infusions were correlated with the intensity of anxiety experienced (Lim et al. 2011). Among ovarian cancer patients, younger age groups were also disproportionately affected by anxiety. Following treatment for ovarian cancer, psychopathology tended to persist, with almost half (47%) of individuals experiencing anxiety symptoms at three months post‐treatment (Arden‐Close et al. 2008).

Long‐term cancer survivors and their spouses also experience elevated levels of anxiety. In a global meta‐analysis of outpatient clinic, hospital, and population‐based samples (Mitchell et al. 2013), the prevalence of anxiety in individuals who had been diagnosed with cancer at least 2 years previously was found to be much higher than in healthy controls (17.9% [95% CI: 12.8%, 23.6%] and 13.9% [95% CI: 9.8%, 18.5%], respectively). Further, almost half (40.1% [95% CI: 25.4%, 55.9%]) of spouses of long‐term cancer survivors developed anxiety. No age or sex effects were reported.

#### Respiratory disease

Three reviews focusing on anxiety in the context of respiratory disease indicated that the prevalence of anxiety was high among adults with COPD (32–57%) (Janssen et al. 2008), and higher still among those with far‐advanced, end‐stage respiratory disease (51–75%) (Solano et al. 2006). Among acute lung injury/acute respiratory distress syndrome (ALI/ARDS) survivors discharged from intensive care units in the United States and Germany, anxiety levels ranged from 23% to 48% (Davydow et al. 2008). No age or sex effects were reported.

#### Diabetes

Three systematic reviews assessed anxiety in adults with diabetes. One high‐quality global review of mostly North American and European studies (Smith et al. 2013) showed that the prevalence is significantly elevated in those with diabetes compared to other groups, but is also dependant on caseness criteria. Approximately 15% to 73% of people with diabetes have anxiety symptoms not meeting threshold criteria (vs. 19.9% to 43.1% of nondiabetic individuals), while 1.4% to 15.6% of people with diabetes meet threshold criteria for an anxiety disorder (vs. 1.6% to 8.8% of nondiabetic individuals). In another review capturing studies predominantly conducted in primary care or clinical settings, women with diabetes were found to have an almost two‐fold higher prevalence of anxiety than men with diabetes (55.3% and 32.9%) (Grigsby et al. 2002). Age effects were not reported. The anxiety disorders that are most common in the context of diabetes are anxiety not otherwise specified, specific phobia, GAD, and social phobia (Grigsby et al. 2002; Clarke and Currie 2009).

#### Other chronic physical diseases

Four reviews assessed anxiety in population subgroups with polycystic ovary syndrome (PCOS), benign joint hypermobility syndrome, musculoskeletal pain, and age‐related macular degeneration. Clinical, mostly Western samples of women with polycystic ovary syndrome (PCOS) had a much higher prevalence of generalized anxiety symptoms than control groups (20.4% and 3.9%, respectively) (Dokras et al. 2012). There is some evidence that social phobia and OCD are comorbid with PCOS. Differences in anxiety levels according to age were not found (Dokras et al. 2012).

Widely varying anxiety prevalence figures have been reported for Mediterranean populations with benign joint hypermobility syndrome (BJHS) (5–68%) (Smith et al. 2014), as well as for Western populations with musculoskeletal pain (0–20.9%) (Andersen et al. 2014). In relation to the latter group, the link between fibromyalgia and anxiety appears to be particularly strong. In people with BJHS, commonly occurring comorbidities are agoraphobia and panic disorder (Smith et al. 2014). The only chronic condition that has failed to show a link with anxiety is age‐related macular degeneration; while this review recruited patients from clinics, it was largely based on US studies (Dawson et al. 2014).

#### Other chronic physical diseases in end‐stage

Four reviews assessed anxiety in end‐stage conditions. A global meta‐analysis of mostly Western studies (Mitchell et al. 2011) estimated the pooled prevalence of anxiety disorders in palliative cancer patients to be 9.8% (95% CI: 6.8%, 13.2%). Estimates appear to vary widely by condition. Among patients with chronic renal failure, the prevalence of anxiety symptoms was found to be 25% in the terminal stage (Janssen et al. 2008), whereas another review found a prevalence of 38% in patients with end‐stage renal disease (Murtagh et al. 2007). Although patients suffering from end‐stage AIDS showed a high symptom prevalence of 8–34%, the highest estimates were found for end‐stage COPD (51–75%) and cancer patients (13–79%) (Solano et al. 2006). No associations between age or sex and anxiety were found in palliative‐care settings (Mitchell et al. 2011).

### Trauma

Three reviews tackled the issue of anxiety in the context of trauma. The first was primarily based on findings from UK and US studies and focused on traumatic limb amputees, and included veterans that had served in Vietnam, Iraq and Afghanistan (Mckechnie and John 2014). Very high prevalence figures were found, with anxiety affecting a fourth of traumatic limb amputees in some studies to over half in others. The second review was global in scope and assessed the frequency of lifetime anxiety among individuals with a history of sexual abuse (Chen et al. 2010). Widely varying anxiety estimates were reported by this review, ranging from 2% to 82%. Finally, a third review focused on GAD in refugees resident in high‐income Western countries; over half of the refugees were from southeast Asia. This meta‐analysis estimated that 4% of refugees experience GAD (Fazel et al. 2005). No age or sex effects in relation to anxiety disorders were reported.

### Vulnerable population subgroups

#### Older people and their caregivers

Five reviews assessed anxiety in older people and their caregivers. The prevalence of anxiety disorders in old age varies widely in community (1.2–14%) and clinical (1–28%) studies conducted mostly in European and North American settings. Estimates are even higher when anxiety symptoms are accounted for. GAD is the commonest anxiety disorder in old age, with the prevalence ranging from 1.3% to 4.7% (Bryant et al. 2008). A random‐effects model (Volkert et al. 2013) showed that specific phobia also occurs frequently in older samples living in the community, while agoraphobia is the rarest anxiety disorder (Bryant et al. 2008). Women are at higher risk for psychopathology than men (Bryant et al. 2008).

Older population subgroups with cognitive dysfunction and their caregivers are disproportionately affected by anxiety (Monastero et al. 2009). In older people with mild cognitive impairment (MCI), the prevalence of anxiety symptoms varies from 11% to 75% (Monastero et al. 2009; Yates et al. 2013). Caregivers of older people with cognitive impairment are also affected by anxiety (prevalence estimates of 3.7–76.5%), with women and younger caregivers showing elevated levels (Cooper et al. 2007; Bryant et al. 2008).

#### Pregnant women

Three reviews focused on pregnant women. A meta‐analysis of international studies (Russell et al. 2013) reported higher OCD prevalence in pregnant (2.07%, [95% CI: 1.26%, 3.37%]) and postpartum (up to 12 months) (2.43%, [95% CI: 1.46%, 4.00%]) women compared to the general population (1.08%, [95%: 0.80%, 1.46%]). Asia and Europe had the lowest prevalence of OCD across conditions, while the Middle East and Africa had the highest. In Ethiopian and Nigerian samples recruited from health clinics and the community (Sawyer et al. 2010), the prevalence of anxiety was found to be high during both the pre‐ and post‐natal periods (14.8% [95% CI: 12.3%, 17.4%] and 14.0% [95% CI: 12.9%, 15.2%], respectively), with younger women showing elevated anxiety compared to older women (Sawyer et al. 2010). There is also some evidence from UK and US studies that a high BMI may contribute to anxiety symptoms during pregnancy (Molyneaux et al. 2014).

#### Individuals identifying as lesbian, gay or bisexual, and self‐harm patients

Two reviews focused on (1) predominantly Western individuals living in the community and identifying as lesbian, gay or bisexual (LGB), and (2) self‐harm patients presenting to general hospitals in countries across the globe. In LBG men, anxiety prevalence was estimated to be 3–20%, while LGB women showed somewhat higher estimates, at 3–39% (King et al. 2008). In a global meta‐analysis of self‐harm patients presenting to hospitals, the prevalence of anxiety disorders was found to be 35% (95% CI: 21.9%, 48.6%). Age‐ and sex‐based differences were small, while rates of anxiety were highest in young and old age groups of self‐harm adults (Hawton et al. 2013). All non‐Western studies of self‐harm patients were based in Asia, while most of the Western studies were conducted in the United Kingdom.

## Discussion

We have synthesized 48 reviews on prevalence studies conducted across the globe. This is the first review to undertake a comprehensive synthesis of the systematic reviews conducted to date on the prevalence of anxiety disorders. It provides a comprehensive, up‐to‐date summary of the state of knowledge in this area.

A number of studies within the reviews were conducted in North America (predominantly the United States) and Europe (Fazel et al. 2005; Cooper et al. 2007; Arden‐Close et al. 2008; Davydow et al. 2008; Fajutrao et al. 2009; Lorains et al. 2011; Mitchell et al. 2011; Fischer et al. 2012; Tully and Cosh 2013; Volkert et al. 2013; Amerio et al. 2014; Goldner et al. 2014; Haller et al. 2014; Mckechnie and John 2014; Molyneaux et al. 2014; Marrie et al. 2015), included clinical and general population samples (Mirza and Jenkins 2004; Bryant et al. 2008; Fajutrao et al. 2009; Monastero et al. 2009; Chen et al. 2010; Sawyer et al. 2010; Lim et al. 2011; Campbell Burton et al. 2013; Hawton et al. 2013; Mitchell et al. 2013; Russell et al. 2013; Yates et al. 2013; Amerio et al. 2014; Haller et al. 2014; Molyneaux et al. 2014), and used mainly DSM or ICD criteria to ascertain diagnoses (Fajutrao et al. 2009; Hawton et al. 2013; Mitchell et al. 2013; Amerio et al. 2014; Goldner et al. 2014; Mckechnie and John 2014; Baxter et al. 2013; Swets et al. 2014). Younger age groups (Arden‐Close et al. 2008; Sawyer et al. 2010; Lim et al. 2011; Webster et al. 2012; Hawton et al. 2013; Yates et al. 2013; Baxter et al. 2014; Haller et al. 2014; Ho et al. 2014; Baxter et al. 2013), women (Somers et al. 2006; Bryant et al. 2008; Clarke and Currie 2009; Webster et al. 2012; Baxter et al. 2013, 2014; Hawton et al. 2013; Yates et al. 2013; Haller et al. 2014; Steel et al. 2014), and people from North America and North Africa/Middle East (Somers et al. 2006; Baxter et al. 2014) showed the highest prevalence of anxiety. Estimates remained stable or declined with age (Somers et al. 2006; Baxter et al. 2013), and secular trends were not observed in relation to the prevalence of total anxiety 4 (Baxter et al. 2014).

Compared to healthy populations or control groups, prevalence was higher in individuals with chronic physical diseases (Mitchell et al. 2013; Yang et al. 2013), and the burden was particularly high in the end stage (Solano et al. 2006; Murtagh et al. 2007; Mitchell et al. 2011). Anxiety symptoms tended to persist post‐disease if present before disease onset (Sawyer et al. 2010), reflecting a chronic, unremitting pattern of psychopathology. Individuals exposed to trauma or who were vulnerable and at risk for stigma (Cooper et al. 2007; Bryant et al. 2008; King et al. 2008; Monastero et al. 2009; Sawyer et al. 2010; Hawton et al. 2013; Russell et al. 2013; Volkert et al. 2013; Yates et al. 2013; Molyneaux et al. 2014), such as older people with cognitive impairment (Yates et al. 2013), were also more likely to experience anxiety. Prevalence figures were heterogeneous, and this made comparison between studies difficult. Heterogeneity was driven by differences in caseness criteria and sampling methods. For example, a meta‐regression (Swets et al. 2014) that assessed the influence of instrument differences on OCD prevalence in the context of schizophrenia showed that the prevalence was higher with the use of the Yale‐Brown Obsessive Compulsive Scale (YBOCS)/Obsessive Compulsive Inventory (OCI) (Goodman et al. 1989; Foa et al. 1998) compared to other instruments. Also, the lower the threshold of the YBOCS, the higher the estimated prevalence. A range of methods was used to measure anxiety, such as, standardized, structured interviews administered by trained professionals, clinician diagnoses, symptom checklists, and self‐report. Some reviews attempted to handle the assessment of anxiety in alternative ways. For example, one review (Baxter et al. 2013) mapped estimates onto ICD or DSM diagnostic criteria and conducted a meta‐analysis to provide an aggregate measure of anxiety. Other reviews either did not attempt a meta‐analysis, or because of very large differences in sampling methods within primary studies, reported disaggregated estimates and ranges found in primary studies. Across reviews, higher prevalence figures were found when subthreshold disorders or symptoms were assessed (Bryant et al. 2008; Goldner et al. 2014; Haller et al. 2014; Swets et al. 2014; Marrie et al. 2015) and when lifetime rather than past‐year or current prevalence was estimated (Somers et al. 2006; Volkert et al. 2013). With the exception of one review (Monastero et al. 2009), authors did not account for the use of psychoactive prescription medicines, such as anxiolytics, which could influence the reporting of anxiety symptoms.

Reviews produced inflated prevalence estimates with the use of less robust methodologies. Within reviews, low and variable response rates across primary studies were identified as another limitation (Somers et al. 2006; Haller et al. 2014). In one review, response rates across studies ranged from 45.9% to 99.5% (Steel et al. 2014).

The areas that received the most attention in the anxiety field include addiction and chronic physical diseases (mainly cancer, CVD, and respiratory diseases), while anxiety disorders other than PTSD in the context of (1) trauma and (2) psychiatric or neurological conditions, such as internet addiction and multiple sclerosis, are relatively new and underresearched areas. Surprisingly, only one review (King et al. 2008) examined LGB groups, despite this population being at high risk for poor health (Fredriksen‐Goldsen et al. 2013). Authors of this review (King et al. 2008) called for further research to produce more refined and consistent definitions of LGB and the recruitment of more representative samples.

Although most of the reviews included in this systematic review were conducted in the last few years, the field of anxiety is rapidly gaining research interest. Some differences in findings and methodologies between older and more recent reviews were noted. For example, recent reviews are increasingly recognizing that early adulthood is the period with the highest peak in anxiety (Arden‐Close et al. 2008; Sawyer et al. 2010; Lim et al. 2011; Webster et al. 2012; Hawton et al. 2013; Yates et al. 2013; Baxter et al. 2014; Haller et al. 2014; Ho et al. 2014; Baxter et al. 2013), and the contexts within which psychopathology is assessed are expanding to a greater number of physical diseases and newly emergent disorders [e.g. internet addiction (Ho et al. 2014)]. Also, newer research is starting to expand its scope to Asian countries (Yang et al. 2013; Ho et al. 2014), a previously identified limitation. More recent reviews are of higher quality, and have started considering instrument differences and their effects on prevalence estimates (Lorains et al. 2011; Swets et al. 2014), another previously identified limitation.

### Recommendations for future research and clinical practice

Recommendations for future research were made by review authors, such as the use of longitudinal designs to address temporality issues (Murtagh et al. 2007; Arden‐Close et al. 2008; Bryant et al. 2008; Janssen et al. 2008; King et al. 2008; Clarke and Currie 2009; Sawyer et al. 2010; Dokras et al. 2012; Fischer et al. 2012; Webster et al. 2012; Russell et al. 2013; Smith et al. 2013; Goldner et al. 2014; Ho et al. 2014; Mckechnie and John 2014); population‐based research that is less susceptible to the help‐seeking/self‐selection bias often present in clinical studies (Grigsby et al. 2002; Murtagh et al. 2007); and the use of valid and reliable instruments and consistent approaches to examine anxiety levels pre‐ and post‐disease (Davydow et al. 2008; Monastero et al. 2009; Sawyer et al. 2010; Webster et al. 2012; Campbell Burton et al. 2013; Smith et al. 2013; Volkert et al. 2013; Goldner et al. 2014; Molyneaux et al. 2014; Swets et al. 2014; Marrie et al. 2015). The measure of “total” or “any anxiety” is not clinically meaningful and is discouraged in favor of the assessment of individual disorders (Smith et al. 2013; Tully and Cosh 2013). Consensus on definitions used to define study samples (e.g., sexual orientation) (King et al. 2008; Fischer et al. 2012; Ho et al. 2014) and diagnostic standardization with respect to the measurement of psychiatric disorders were also emphasized (Monastero et al. 2009; Goldner et al. 2014; Swets et al. 2014), as well as research into the risk factors, illness trajectory, hereditary, and biological markers of anxiety (Somers et al. 2006; Davydow et al. 2008; Monastero et al. 2009; Chen et al. 2010; Dokras et al. 2012; Russell et al. 2013; Amerio et al. 2014; Ho et al. 2014; Smith et al. 2014), and the appropriateness of anxiety screening measures in the context of physical diseases and cultures around the world (who may express distress differently) (Fazel et al. 2005; Bryant et al. 2008; Sawyer et al. 2010; Baxter et al. 2013; Hawton et al. 2013; Steel et al. 2014). Research questions should be structured around theories (Arden‐Close et al. 2008; Webster et al. 2012). Recommendations were made for the inclusion of appropriate control subjects in studies to determine whether prevalence differs between exposed and comparison groups (Yang et al. 2013; Dawson et al. 2014). Finally, further treatment or intervention studies are needed to alleviate anxiety (Mirza and Jenkins 2004; Murtagh et al. 2007; Arden‐Close et al. 2008; Clarke and Currie 2009; Fatseas et al. 2010; Lim et al. 2011; Amerio et al. 2014; Goldner et al. 2014; Haller et al. 2014; Ho et al. 2014; Smith et al. 2014; Swets et al. 2014).

Clinical recommendations included the administration of targeted anxiety screening and, if necessary, treatment. For example, suggestions were made for the screening of substance users at treatment entry (Fatseas et al. 2010) or patients with noncardiac chest pain presenting to acute care (Webster et al. 2012). It was also shown that certain anxiety disorders were more common in certain groups, such as OCD in schizophrenia (Swets et al. 2014), PD and GAD in CVD (Campbell Burton et al. 2013), and SP in diabetes (Grigsby et al. 2002). Additional research on individual anxiety disorders is needed to confirm these findings, but once this is underway, further impetus will be provided for the targeted screening of high‐risk groups in relation to individual anxiety disorders.

This review has some limitations. Despite extensive database searches, it is possible that some reviews have been missed. Also, the high heterogeneity in anxiety assessment methods and sampling frameworks within primary studies contributed to large differences in prevalence estimates within and across reviews, making it difficult to draw conclusions about the burden of anxiety. Also, a number of the reviews were based on English‐language studies conducted in predominantly Western settings, making generalizability to other parts of the world difficult.

## Conclusions

Anxiety disorders are increasingly being recognized as important determinants of poor health and major contributors to health service use across the globe (Nutt and Ballenger 2003; Simpson et al. 2010). Despite epidemiologic advances in this field, important areas of research remain under‐ or unexplored. There is a need for further studies on the prevalence of anxiety disorders in the context of: personality disorders; Indigenous cultures in Canada, the United States, New Zealand, and Australia; African, Middle Eastern, Eastern European, Asian and South American countries; and marginalized populations, such as injection drug users, street youth, and sex workers. These recommendations can serve to guide the research agenda, and most importantly, help develop tailored and timely interventions.

## Conflict of interest

None declared.

## Appendix 1 Checklist of items to include when reporting a systematic review or meta‐analysis.

**Table:**

Section/Topic	No.	Checklist item
Title		
Title	1	Identify the report as a systematic review, meta‐analysis, or both
Structured summary	2	Provide a structured summary including, as applicable: background; objectives; data sources; study eligibility criteria, participants, and interventions; study appraisal and synthesis methods; results; limitations; conclusions and implications of key findings; systematic review registration number
Introduction		
Rationale	3	Describe the rationale for the review in the context of what is already known
Objectives	4	Provide an explicit statement of questions being addressed with reference to participants, interventions, comparisons, outcomes, and study design (PICOS)
Methods		
Protocol and registration	5	Indicate if a review protocol exists, if and where it can be accessed (e.g., Web address), and, if available, provide registration information including registration number
Eligibility criteria	6	Specify study characteristics (e.g., PICOS, length of follow‐up) and report characteristics (e.g., years considered, language, publication status) used as criteria for eligibility, giving rationale
Information sources	7	Describe all information sources (e.g., databases with dates of coverage, contact with study authors to identify additional studies) in the search and date last searched
Search	8	Present full electronic search strategy for at least one database, including any limits used, such that it could be repeated
Study selection	9	State the process for selecting studies (i.e., screening, eligibility, included in systematic review, and, if applicable, included in the meta‐analysis)
Data collection process	10	Describe method of data extraction from reports (e.g., piloted forms, independently, in duplicate) and any processes for obtaining and confirming data from investigators
Data items	11	List and define all variables for which data were sought (e.g., PICOS, funding sources) and any assumptions and simplifications made
Risk of bias in individual studies	12	Describe methods used for assessing risk of bias of individual studies (including specification of whether this was done at the study or outcome level), and how this information is to be used in any data synthesis
Summary measures	13	State the principal summary measures (e.g., risk ratio, difference in means)
Synthesis of results	14	Describe the methods of handling data and combining results of studies, if done, including measures of consistency (e.g., I2) for each meta‐analysis
Risk of bias across studies	15	Specify any assessment of risk of bias that may affect the cumulative evidence (e.g., publication bias, selective reporting within studies)
Additional analyses	16	Describe methods of additional analyses (e.g., sensitivity or subgroup analyses, meta‐regression), if done, indicating which were pre‐specified
Results		
Study selection	17	Give numbers of studies screened, assessed for eligibility, and included in the review, with reasons for exclusions at each stage, ideally with a flow diagram
Study characteristics	18	For each study, present characteristics for which data were extracted (e.g., study size, PICOS, follow‐up period) and provide the citations
Risk of bias within studies	19	Present data on risk of bias of each study and, if available, any outcome‐level assessment (see Item 12)
Results of individual studies	20	For all outcomes considered (benefits or harms), present, for each study: (1) simple summary data for each intervention group and (2) effect estimates and confidence intervals, ideally with a forest plot
Synthesis of results	21	Present results of each meta‐analysis done, including confidence intervals and measures of consistency
Risk of bias across studies	22	Present results of any assessment of risk of bias across studies (see Item 15)
Additional analysis	23	Give results of additional analyses, if done (e.g., sensitivity or subgroup analyses, meta‐regression [see Item 16])
Discussion		
Summary of evidence	24	Summarize the main findings including the strength of evidence for each main outcome; consider their relevance to key groups (e.g., health care providers, users, and policy makers)
Limitations	25	Discuss limitations at study and outcome level (e.g., risk of bias), and at review level (e.g., incomplete retrieval of identified research, reporting bias)
Conclusions	26	Provide a general interpretation of the results in the context of other evidence, and implications for future research
Funding		
Funding	27	Describe sources of funding for the systematic review and other support (e.g., supply of data); role of funders for the systematic review

## Appendix 2 Search terms

### Embase

1. exp Meta Analysis/
2. ((meta adj analy$) or metaanalys$).tw.
3. (systematic adj (review$1 or overview$1)).tw.
4. or/1–3
5. cancerlit.ab.
6. cochrane.ab.
7. embase.ab.
8. (psychlit or psyclit).ab.
9. (psychinfo or psycinfo).ab.
10. (cinahl or cinhal).ab.
11. science citation index.ab.
12. bids.ab.
13. or/5–12
14. reference lists.ab.
15. bibliograph$.ab.
16. hand‐search$.ab.
17. manual search$.ab.
18. relevant journals.ab.
19. or/14–18
20. data extraction.ab.
21. selection criteria.ab.
22. 20 or 21
23. review.pt.
24. 22 and 23
25. letter.pt.
26. editorial.pt.
27. animal/
28. human/
29. 27 not (27 and 28)
30. or/25–26, 29
31. 4 or 13 or 19 or 24
32. 31 not 30
33. anxiety/or generalized anxiety disorder/or anxiety disorder/
34. prevalence.mp.
35. 32 and 33 and 34
36. prevalen*.mp. [mp = title, abstract, subject headings, heading word, drug trade name, original title, device manufacturer, drug manufacturer, device trade name, keyword]
37. 32 and 33 and 36

### Medline

1. Meta‐Analysis as Topic/
2. meta analy$.tw.
3. metaanaly$.tw.
4. Meta‐Analysis/
5. (systematic adj (review$1 or overview$1)).tw.
6. exp Review Literature as Topic/
7. or/1–6
8. cochrane.ab.
9. embase.ab.
10. (psychlit or psyclit).ab.
11. (psychinfo or psycinfo).ab.
12. (cinahl or cinhal).ab.
13. science citation index.ab.
14. bids.ab.
15. cancerlit.ab.
16. or/8–15
17. reference list$.ab.
18. bibliograph$.ab.
19. hand‐search$.ab.
20. relevant journals.ab.
21. manual search$.ab.
22. or/17–21
23. selection criteria.ab.
24. data extraction.ab.
25. 23 or 24
26. Review/
27. 25 and 26
28. Comment/
29. Letter/
30. Editorial/
31. animal/
32. human/
33. 31 not (31 and 32)
34. or/28–30, 33
35. 7 or 16 or 22 or 27
36. 35 not 34
37. exp Anxiety/or exp Anxiety Disorders/
38. 36 and 37
39. prevalence.mp.
40. 36 and 37 and 39
41. 37 and 39
42. 36 and 41
43. prevalen*.mp. [mp = title, abstract, original title, name of substance word, subject heading word, keyword heading word, protocol supplementary concept word, rare disease supplementary concept word, unique identifier]
44. 36 and 37 and 43

### PsycInfo

1. exp Meta Analysis/
2. meta analy$.tw.
3. metaanaly$.tw.
4. (systematic adj –n – (review$1 or overview$1)).tw.
5. exp “Literature Review”/
6. or/1–5
7. cochrane.ab.
8. embase.ab.
9. (psychlit or psyclit).ab.
10. (cinahl or cinhal).ab.
11. science citation index.ab.
12. bids.ab.
13. cancerlit.ab.
14. reference list$.ab.
15. bibliograph$.ab.
16. hand‐search$.ab.
17. relevant journals.ab.
18. manual search$.ab.
19. or/14–18
20. selection criteria.ab.
21. data extraction.ab.
22. 20 or 21
23. exp “Literature Review”/
24. 22 and 23
25. comment/
26. letter/
27. editorial/
28. human.po.
29. animal.po.
30. (animal not (human and animal)).po.
31. 25 or 26 or 27 or 30
32. prevalence.mp. [mp = title, abstract, heading word, table of contents, key concepts, original title, tests & measures]
33. exp Anxiety Disorders/or exp Anxiety/
34. 6 or 19 or 24
35. 32 and 33 and 34
36. 35 not 31

## Appendix 3

**Table A1:** Systematic reviews describing the prevalence of anxiety disorders

Review details	Population characteristics and sample size	Sampling methods	Anxiety assessment methods	Anxiety prevalence (prevalence %, [95% CI]) and summary of results
**Global distribution of anxiety disorders**				
Somers 2006 *Search:* 2004 *# incl. studies* 39 *Meta‐analysis:* yes	Adults Range: 500–20,000	Community surveys using probability sampling	Diagnostic criteria, standardized instruments or clinician diagnosis	Pooled one‐year and lifetime prevalence of: Total anxiety disorders: 10.6% (7.5, 14.3), 16.6% (12.7, 21.1) PD: 1.0% (0.6, 1.5), 1.2% (0.7, 1.9) Agoraphobia: 1.6% (1.0, 2.3), 3.1% (2.1, 4.4) SAD: 4.5% (3.0, 6.4), 2.5% (1.4, 4.0) SP: 3.0% (1.0, 5.8) and 4.9% (3.4, 6.8) OCD: 0.5% (0.3, 0.9), 1.3% (0.9, 1.8) GAD: 2.6% (1.4, 4.2), 6.2% (4.0, 9.2) Anxiety higher in women SAD rates decline with age Switzerland, US: 23–28.7; Korea: 9.2
Baxter 2013 *Search:* 2009 *# incl. studies* 87 *Meta‐analysis:* yes	44 countries across the globe Median: 2419	Community samples	Interview schedules, semi‐structured instruments, diagnostic instruments that mapped to DSM or ICD	Global prevalence: 7.3% (4.8–10.9) 5.3% (3.5, 8.1) in African & 10.4% (7.0, 15.5) in Euro/Anglo cultures Women 2× men; younger people more affected Adults 55 + 20% less anxiety than 35–55 20–50% lower risk in cultures compared to Euro/Anglo
Mirza 2004 *Search:* March 2002 *# incl. studies:* 20 *Meta‐analysis:* no	Adults ages 18–65 years from community and clinical settings Range: 113–2620	Population‐based, community, primary care samples; patients presenting to traditional or faith healers; psychiatric outpatients or inpatients Clinical and community settings in Pakistan	Psychiatric diagnoses, diagnoses made by trained workers using validated instruments	Anxiety prevalence: 1.76–25% Middle‐aged more affected
Vehling 2012 *Search: not rep.* *# incl. studies* 89 *Meta‐analysis:* yes	Adults 38–73 years Sample size not rep.	Mostly US studies	Structured clinical interviews	4‐week prev. of anxiety disorders: 10.2% (6.9, 14.8) [International & German]; 13.5% (7.1, 24.3) [German only] Germans with breast cancer: anxiety 28–33%; SP 5.2% (3.3, 8.2) & GAD 3.7% (2.3, 6.0) common
Baxter 2014 *Search:* 2009 *# incl. studies* 91 *Meta‐analysis:* yes	DSM/ICD community studies on people, all ages; GHQ for studies on secular trends Range: 116–78,290	Community‐based studies	Surveys, diagnostic criteria	Age‐standardized global point prev.: 3.8% (3.6–4.1%) in 1990; 4.0% (3.7–4.2%) in 2005 and 2010 Anxiety women:men ratio of 1.9:1 Sharp rise in adolescents; highest prev. 15–35 years Prev. lowest in East Asia [2.8% (2.2–3.4%)] and highest in North America & North Africa/Middle East [7.7%, (6.8–8.8%) vs.7.7% (6.0–10%)]
Haller 2014 *Search:* 2006 *# incl. studies:* 18 *Meta‐analysis:* no	Pop‐based studies of subthreshold DSM/ICD GAD in adults 15–96 years Range: 90–17,739	General population and primary care sample Clinical and community settings Mostly North American and European data	Diagnostic criteria	12‐month median prev. – 3.9% (range: 2.1–6.6%) When GAD duration criterion relaxed, prev of subthreshold GAD increased: 12 month prev. with 3 + mo. vs. 1 + mo. duration: 3.6% vs. 6.1% Higher prev in younger people in clinical samples, but higher in older people in community (3%) Median point prev. in primary care: 5.9% (1.3–8.3%) Women higher prev than men 42% of young women with subthreshold GAD also had other subthreshold mental disorders Subthreshold GAD mostly comorbid with other anxiety disorders
Steel 2014 *Search:* Jan 2014 *# incl. studies* 174 *Meta‐analysis:* yes	26 high‐income and 37 LMIC countries Mostly 16–65 years Samples of 450+ people Median n: 2314	Population sample; Census or probabilistic epidemiological procedures used in surveys Community settings		Period prev of anxiety disorders in men 4.3% (3.7–4.9%), 8.7% (7.7–9.8%) in women Lifetime prev of anxiety disorders in men 10.1% (8.8–11.6%), 18.2% (16.2–20.4%) in women Same pattern of gender differences in HIC and LMIC countries
**Addiction**				
Fatseas 2010 *Search:* Jan. 2009 *# incl. studies* 18 *Meta‐analysis:* no	All‐age participants with opiate dependence Range: 50–716	Clinical samples from drug treatment programs	Structured interviews and diagnostic criteria	Lifetime prev: 2–58% and 5–67% SP, SAD, GAD common Narrower prev with recent DSM criteria
Fischer 2012 *Search:* Dec. 2011 *# incl. studies* 9 *Meta‐analysis:* yes	Adults Range: 1,086–166,453	General population samples Community settings All North American, mostly US studies	Standardized (clinical diagnostic) and nonstandardized indicators or symptoms	Symptoms prev in general pop: 16% (1–30)
Goldner 2014 *Search:* April 2012 *# incl. studies* 11 *Meta‐analysis:* yes	Patients at admission or in treatment for substance abuse problems from US and Canada Sample size not rep.	Chart review of admissions and discharges, survey of people entering treatment programs Clinical settings All North American, mostly US studies	Clinical diagnostics based on DSM, other clinical assessments, or symptom self‐reports	Prev of diagnosis and symptoms: 38% (14–63) Diagnosis prev: 29 (14–44); symptoms: 50% (16–84) No significant age or sex‐effects
Lorains 2011 *Search:* Sept. 2010 *# incl. studies* 11 *Meta‐analysis:* yes	Adults Range: 2417–43,093	General population samples/surveys Community settings Mostly US studies	Validated screening tool/standardized measurement tools	Prev: 37.4%
Ho 2014 *Search:* 2012 *# incl. studies* 8 *Meta‐analysis:* yes	Age 10+ 1641 patients with internet addition (IA) and 11,210 controls without IA	Postal survey, students, respondents to ads Community settings Mostly Asian samples	Standard questionnaires, symptom checklists, interviews	Prev of anxiety in IA patients vs normal controls: 23.3% (95% CI: 14.8–34.8) vs 10.3% (5.0–19.9) Anxiety most prev in young age groups with IA (19–39 years highest burden)
**Other mental and neurological disorders**				
Fajutrao 2009 *Search:* past 10 years *# incl. studies* 26 *Meta‐analysis:* no	Patients with bipolar disorder Range: 72–1,631,462	Surveys; general population, inpatients Clinical and community settings European studies	DSM diagnoses	13–28% of bipolar patients with anxiety GAD and PD common 70%, 24%, 16% for Italy, France, Germany
Amerio 2014 *Search:* Mar 2013 *# incl. studies:* 64 *Meta‐analysis:* no	Pop‐based and hospital‐based studies on DSM OCD in bipolar disorder (BD), ages 6 + Range: 15–1416	Clinical and community settings Most studies conducted in Europe and North America	Interviews, DSM criteria	Pop‐based US, Italian studies: lifetime prev of OCD in BD: 11.1–21% Hospital‐based studies: lifetime prev: 1.8–35.1% OCD onset usually concomitant with first mood episode
Swets 2014 *Search:* Dec 2009 *# incl. studies* 43 *Meta‐analysis:* yes	Schizophrenia patients 18–509	Mainly clinical settings	Interviews, symptom scales, DSM	Prev of OCD and OCS in schizo. – 12.3% (9.7–15.4%) & 30.7% (23–39.6%); meta‐regression: prev of OCS: 30.3% Lower OCD prev: Sub‐Saharan African origin, recent onset schizo. Higher OCD prev: DSM‐IV and Y‐BOCS; after adjustment: OCD prev 13.6% (11.8–15.8%) Higher prev with Y‐BOCS, OCI Prev of OCD/OCS in studies using YBOCS/OCI : 16.9% (13.25–21.1%) vs studies not using YBOCS/OCI: 8.0 (5.3–11.9%) Higher the YBOCS threshold, lower OCS prev
Marrie 2015 *Search:* Nov. 2013 *# incl. studies* 118 *Meta‐analysis:* yes	MS populations; all ages Range: not rep.	Population‐based, possibly other sampling Some studies conducted in community settings Most studies from Central or Western Europe or parts of North America	Structured diagnostic interviews, medical records review, self‐reported diagnoses, validated instruments	Prev. of anxiety disorders & symptoms in MS: 31.7% vs 63.4%; Higher anxiety in MS than in controls Anxiety at MS symptom onset: 2.72% vs 6.23% at diagnosis; prev. of health anxiety in MS: 26.4% Pop‐based studies – anxiety prev: 21.9% (8.76–35.0%) Anxiety prev questionnaires vs admin data/medical records: 25.5% (16.7–34.3) vs. 15.4% (0–39.0)
**Chronic physical diseases**				
*Cardiovascular disease*				
Janssen 2008 *Search:* 2007 *# incl. studies* 39 *Meta‐analysis:* no	End‐of‐life CHF, COPD, CRF patients Mean age: 38–86 Sample size: not rep.	Proxies and patients recruited, chart/medical record review		CHF: 2–49% (anxiety prev) COPD: 32–57% CRF: 20–41% CRF terminal: 25%
Solano 2006 *Search:* June 2004 *# incl. studies* 64 *Meta‐analysis:* no	Adults with advanced cancer, AIDS, heart disease, COPD, renal disease Range: 19–10,379	Medical records, interviews with patients’ families, proxies used, prescriptions for psychotropic drugs Some studies conducted in clinical settings		Prev of anxiety symptoms: Cancer: 13–79% AIDS: 8–34% Heart disease: 49% COPD: 51–75% Renal disease: 39–70%
Tully 2013 *Search:* May 2011 *# incl. studies* 12 *Meta‐analysis:* yes	Older people: median age: 60 years Range: 86–1015	Primary care sample, CHD patients attending rehab, outpatient clinic, people going in for surgery Clinical studies Mostly US studies	Diagnostic interview tools	GAD prevalence: 10.94% (7.8, 14.0) Lifetime GAD: 25.8% (20.84, 30.8)
Clarke 2009 *Search:* May 2003 *# incl. studies* 159 *Meta‐analysis:* no	Sample size: not rep.			Heart disease – PD: 10–50% Diabetes mellitus: 14% with GAD Cancer: 15–23%; more advanced stage: 69% Arthritis and osteoporosis link to anxiety Women more anxiety than men (55.3% vs 32.9%)
Webster 2012 *Search:* Nov. 2010 *# incl. studies* 12 *Meta‐analysis:* no	Adults with (nonspecific) acute chest pain in acute care Range: 50–1300	Patients admitted to ED Clinical studies	Symptom checklists	21–53.5% of NCCP patients had probable anxiety Women and younger patients – elevated anxiety Anxiety levels in NCCP similar to or higher than in CCP or healthy controls
Campbell Burton 2013 *Search:* March 2011 *# incl. studies* 44 *Meta‐analysis:* yes	Mean age: 66–71 years Range: 15–498	Population‐based (all stroke patients recruited from particular geographical area), hospital‐ and rehabilitation‐based (inpatients or those attending rehab facilities), community‐based (did not attempt to capture all stroke cases in geographic area) Clinical and community settings	Anxiety symptom scales, clinical diagnoses, single question measure, researcher‐developed questions	Prev of anxiety disorders: 18% (8–29) PD & GAD common Anxiety caseness (rating scales): 25% (21–28) 1/3 of patients with post‐stroke anxiety had pre‐stroke mood or anxiety High anxiety‐depression comorbidity
*Cancer*				
Clarke 2009 – previously described				
Solano 2006 – previously described				
Yang 2013 *Search:* Sep. 2012 *# incl. studies* 17 *Meta‐analysis:* yes	Adults 18 + years from Mainland China Range: 380–2554	Unclear (assessed “patients”) Mainland China studies	Clinical diagnosis, symptom checklists, self‐report questionnaires	Anxiety prev: 49.7% (range: 20–89.1) in cancer, and 17.50% in the noncancer control group
Vehling 2012 – previously described				
Lim 2011 *Search:* 2010 *# incl. studies* 10 *Meta‐analysis:* no	Patients 21–65 on treatment for early‐stage breast cancer Range: 48–332	Women who were undergoing/had undergone breast cancer treatment (ex. RCT studies: patients from the center randomly selected to receive various treatment types; non‐RCT studies: women undergoing various cancer treatments/surgeries, patients from oncology clinics; patients assessed at home) Clinical and community settings	Symptom checklists	20% to 58% Less anxiety if given treatment choice More state/trait anxiety during chemo than radiotherapy Greater trait anxiety in young women during chemo
Arden‐Close 2008 *Search:* May 2007 *# incl. studies* 18 *Meta‐analysis:* no	Ovarian cancer patients Range: 9–246	Unclear (included patients, cancer survivors) Mostly US studies	Standardized and nonstandardized assessment tools, symptom checklists	Prev: 47% at 3 months following treatment Anxiety levels increased from treatment completion date to 3‐month follow‐up Young age groups disproportionately affected
Mitchell 2013 *Search:* March 2013 *# incl. studies* 43 *Meta‐analysis:* yes	Adult patients compared with spouses, IQR sample size: 145–270 Adult patients and healthy controls IQR:1328–25,245	Cases: outpatient clinic, database/cancer registry, hospitals, general population; recruitment: random sample (population‐based), patients treated in a certain time period; prescription for psychotropic drugs; Controls: comparator matching by sociodemographics, convenience sample, matched partner pair Clinical and community settings	Symptom checklists, structured questionnaire for DSM, prescription of psychotropic drugs, clinical diagnosis	Prev. long‐term cancer survivors vs. healthy controls: 17.9% (12.8–23.6), 13.9% (9.8–18.5); anxiety higher in cancer patients regardless of methodological factors Long‐term cancer survivors vs. spouses: 28% (22.3–33.9), 40.1% (25.4–55.9); age/sex effect not rep.
*Respiratory disease*				
Janssen 2008 – previously described				
Solano 2006 – previously described				
Davydow 2008 *Search:* April 2007 *# incl. studies* 10 *Meta‐analysis:* no	Adult survivors in the United States and Germany 321 patients	Sampling not mentioned – assessed patients following ICU discharge US and German studies	Symptom checklists	23–48%
*Diabetes*				
Smith 2013 *Search:* July 2012 *# incl. studies* 12 *Meta‐analysis:* yes	Adults ages 16 + years Range: 635–217,379	Sampling not mentioned/unclear Mostly North American and European studies	Surveys, clinical interview(s), validated scale	Prev (HADS‐A): 15–73% in diabetic patients and 19.9–43.1% in ref groups Prev of anxiety disorders (clinical interviews): 1.4–15.6% in diabetic patients; 1.6–8.8% in ref
Grigsby 2002 *Search:* 2001 *# incl. studies* 18 *Meta‐analysis:* yes	Adults ages 18 + Range: 20–634 (for diabetic subjects)	Most studies based on primary care/clinical samples	Structured or semi‐structured diagnostic interviews, self‐report measures	Current and lifetime prev (%) of anxiety in diabetes: GAD: 13.5, 20.5; panic: 1.2, 1.9 OCD: 1.3, 1.1; Agoraphobia: 4.6, 10.2 SP: 21.6, 24.8; SAD: 7.3, 9.3 Any phobia: 6.8, 10.4 Any anxiety disorder: 14.0, 25.8 Anxiety not otherwise specified: 26.5, 39.0 Elevated symptoms: 39.6 Higher prev of anxiety symptoms in women than in men: 55.3 vs. 32.9 No diff by diab. Type; GAD most prevalent Anxiety dis. & symp: 25.8% & 39.6%
Clarke 2009 – previously described				
**Other chronic physical diseases**				
Dokras 2012 *Search:* April 2011 *# incl. studies* 9 *Meta‐analysis:* yes	PCOS subjects and non‐PCOS controls Range: 44–206	Screened clinic populations, 1 study used telephone screening Mostly clinical settings Mostly Western studies	Anxiety screening tool	Anxiety prev: 1–37.5% in PCOS; 0–13 in controls Prev of generalized anxiety symptoms in PCOS and controls: 20.4% vs 3.9% SAD and OCD more common in PCOS; age effects not rep.
Smith 2014 *Search:* January 2013 *# incl. studies* 14 *Meta‐analysis:* yes	Mostly adult, Medi‐terranean pop. 30 BJHS people & 25 controls‐182 people BJHS & 1123 controls	Clinically representative participants Recruited participants from school settings, university, primary care/community health care settings, hospital outpatient departments		Anxiety prev: 5–68% in BJHS; 5–32% in non‐BJHS BJHS have more PD, agoraphobia and fear than non‐BJHS
Andersen 2014 *Search:* Sept. 2012 *# incl. studies* 24 *Meta‐analysis:* no	Adults (mean age: 43–50) from Western countries with musculoskeletal pain >= 3 months Range: 84–3928	Primary care clinics or hospital services; recruitment: general population, through ads.; mostly outpatients Western studies	Symptom checklists and structured clinical interview	Pooled one‐year and lifetime prevalence of: Clinical and general anxiety levels: 0–20.9% (highest prev. with SCID) Highest anxiety prev. in fibromyalgia
Dawson 2014 *Search:* Feb 2012 *# incl. studies* 16 *Meta‐analysis:* no	Adults with age‐related macular degeneration (AMD) age 18 + Range: 51–32,702	Recruited from eye clinics, GP clinics Clinical/specialist setting Western studies, many US	Almost all symptom checklists, structured clinical interview	Generally no link with anxiety found, but one study reported prev of 30.1% in AMD
**Other chronic physical diseases in end‐stage**				
Mitchell 2011 *Search:* Nov. 2010 *# incl. studies* 94 *Meta‐analysis:* yes	4007 adults age 18 + in palliative care; 10,071 adults 18 + in palliative care and oncological settings	Patients from oncological, hematological, and palliative‐care settings Mostly Western studies	Psychiatric interviews	9.8% (6.8–13.2) in palliative‐care, and 10.3% (5.1–17.0) in oncological and hematological settings
Janssen 2008 – previously described				
Murtagh 2007 *Search:* April 2005 *# incl. studies* 60 *Meta‐analysis:* No	Adult patients diagnosis of end‐stage renal disease Range: 19–5256	Clinical settings	Standardized psychiatric interview, survey, validated screening tools	Anxiety prev: 38% (12–52)
Solano 2006 – previously described				
**Trauma**				
Mckechnie 2014 *Search:* June 2013 *# incl. studies* 13 *Meta‐analysis:* no	Traumatic limb amputees, age 18 + Range: NR	Military patients (including veterans from Vietnam, Iraq, Afghanistan) Mostly UK and US studies	ICD or DSM diagnoses, symptom checklists	Anxiety ranged from 25.4–57% in this pop
Chen 2010 *Search:* Dec. 2008 *# incl. studies* 37 studies *Meta‐analysis:* yes	Individuals with history of sexual abuse compared to those without Range: 34–1,574,100	Registries, school health or GP records; referral from the rape crisis center, conscripts, voters, general population, friends of victims (controls) Clinical, community settings	Mostly structured diagnostic interview	Lifetime anxiety in people with sex abuse: 2–82% Associations between sexual abuse and MD persisted regardless of sex of survivor and age at which abuse occurred
Fazel 2005 *Search:* Dec. 2002 *# incl. studies* 20 *Meta‐analysis:* yes	Adult refugees from southeast Asia, former Yugoslavia, middle east, Central America; weighted mean age = 27 6743 adult refugees	Opportunistic sampling (ex. student enrolment lists, health‐screening programs) High‐income western countries; ¾ participants from southeast Asia Community settings	Clinical interview, trained interviewers using validated diagnostic methods	4% (3–6) of refugees diagnosed with GAD
**Vulnerable population subgroups**				
*Older people and their caregivers*				
Bryant 2008 *Search:* 2007 *# incl. studies* 49 *Meta‐analysis:* no	People 60 + years in community or clinical settings Range: 286–10,641	Community surveys, GP lists, geriatric hospital, general hospital, case register, clinic referrals, consecutive series; participants included institutionalized older adults, nursing home residents	Checklists, self‐report, clinical record review, clinical diagnoses	Anxiety in community: 1.2–14%; anxiety in clinical samples: 1–28% Anxiety symptoms: 15–52.3% in community and 15–56% in clinical samples PD: 1.4–25.6%; Agoraphobia: 0.4–20% SP: 5.9–13.1%; SAD: 0.0–18.7% OCD: 0.6–1.8%; PD: 0.0–10.5% GAD commonest & more women with anxiety
Volkert 2013 *Search date:* Dec. 2011 *# incl. studies* 25 *Meta‐analysis:* yes	Older people 50 + years mainly from Germany, US, Sweden Range: 242–22,777	Mostly random samples, representative samples, 1 study contacted all elderly of one town, sample stratification according to various criteria Community settings	Diagnostic interviews, dimensional instruments	Current and lifetime PD: 0.88% (0.76, 0.99), 2.63% (2.43, 2.84) Agoraphobia: 0.53% (0.39, 0.66), 1.00% (0.54, 1.45); SP: 4.52% (4.15, 4.89), 6.66% (6.17, 7.15) SAD: 1.31% (1.18, 1.44), 5.07% (4.82, 5.32) GAD: 2.30% (2.03, 2.57), 6.36% (5.57, 7.14) OCD: 0.90% (0.63, 1.17), 0.97 (0.55, 1.38) Lower SP prev in old
Monastero 2009 *Search:* Aug. 2008 *# incl. studies* 27 *Meta‐analysis:* no	Mean age at baseline ranged from 65–80 years Range: 44–2879	Hospital‐based samples with MCI, population‐based samples with MCI, clinical trial of MCI subjects Clinical and community settings	Behavioral instruments including diagnostic interviews (clinical interview, trained interviewer)	Prev: 11–74% Anxiety is common in Alzheimer's disease
Yates 2013 *Search:* Nov. 2012 *# incl. studies* 18 *Meta‐analysis:* yes	Clinical samples with MCI or community samples of older people Range: 18–6892	People self‐referred or referred by GP to memory clinics; people recruited from general population	Anxiety symptom scales	Prev. of anxiety: 11–75% in elderly with MCI Women and younger caregivers higher anxiety
Cooper 2007 *Search:*2005 *# incl. studies* 33 *Meta‐analysis:* no	Caregivers of people with dementia Range: 34–979	Case‐note review to identify caregivers of old people referred to psychiatry service; cohort studies UK and US studies	Diagnostic interview schedules, symptom scale	3.7–76.5% Prev depended on study time period, sample, anxiety caseness definition
*Pregnant women*				
Russell 2013 *Search:* August 2012 *# incl. studies* 17 *Meta‐analysis:* yes	Pregnant and postpartum women (up to 12 months) Range: 27–3929	Community and outpatient referrals Controls: general population samples All continents included	Structured diagnostic interviews	Overall prev: 1.08% (0.80, 1.46) in general pop of women, 2.07% (1.26, 3.37) during pregnancy, 2.43% (1.46, 4.00) during postpartum
Molyneaux 2014 *Search:* Jan 2013 *# incl. studies* 62 *Meta‐analysis:* insufficient studies for meta‐a for anxiety	Overweight or obese women at start of pregnancy vs normal weight control women Total 540,373 women	Medical records; women seeking prenatal care; primary care or hospital centre sample; all women living in Avon expected to deliver in a certain time period; Recruitment from prenatal exercise classes, obstetrician and gynaecologist waiting rooms (through newsletter), women with low‐income insurance Clinical and community Mostly Western studies (esp. UK and US)	Diagnostic and screening measures; did not include measures of state anxiety	Low‐income Brazilian women: anxiety prev 35% obese, 35.7% overweight, 31% normal weight Postpartum anxiety prev: symptoms across studies ranged from 4.7% in obese (4% in overweight, 4.2% in normal weight) to 33.3% (13.3% in overweight, 16.4% normal weight)
Sawyer 2010 *Search:* January 2009 *# incl. studies* 35 *Meta‐analysis:* yes	Ethiopian and Nigerian women Range: 101–632 (anxiety studies)	Antenatal and postnatal health clinics, community All studies from Africa, most from Nigeria	Most used structured clinical interviews, many used self‐administered measures, some used both	Pre‐ and postnatal anxiety prevalence: 14.8% (12.3–17.4) and 14.0% (12.9–15.2) Younger women more anxious
*LGB and self‐harm patients*				
King 2008 *Search:* 2005 *# incl. studies* 25 *Meta‐analysis:* yes	Anxiety in LGB and heterosexual groups Range: 79–194 (for anxiety studies)	Random sampling, multi‐stage sampling, snowball sampling, some primary studies did not specify method Community settings	Standardized scales	Anxiety prev: 3–20% and 3–39% in men and women Stigma and discrimination contributors
Hawton 2013 *Search:* Nov. 2011 *# incl. studies* 50 *Meta‐analysis:* yes	All age patients presented to hospitals following self‐harm (self‐poisoning, self‐injury, suicide attempt) Range: 22–1158	Consecutive admissions to different departments, recruitment on specific days, consecutive referrals to suicide unit, random sample Clinical samples All studies of non‐Western countries from Asia, most Western studies from UK	Research diagnostic criteria and clinical diagnoses converted to DSM‐IV	Prev of anxiety disorders: 34.6% (21.9–48.6) Anxiety prev in women and men: 42% & 38% Small sex‐based diff.; prev high in young and old

SP, specific or simple phobia; PD, panic disorder; GAD, generalized anxiety disorder; SAD, social anxiety disorder; OCD, obsessive compulsive disorder; anx, anxiety; NR, not reported.

**Table A2:** Directions for future research and reported limitations

Review details	Directions for future research	Reported limitations	QA^a^
**Global distribution of anxiety disorders**			
Somers 2006 *Search:* 2004 *# incl. studies* 39 *Meta‐analysis:* yes	Incidence and onset studies needed Research on anxiety risk & protective factors, and social variables as mediators Prev of anxiety in special groups (e.g., medical patients, residents of nursing homes) Clarify epidemiology of anxiety to help with deployment of treatment	*Original studies* Heterogeneity: diagnosis criteria and instruments used (ex. lower estimates with use of DIS and DSM‐III than CIDI and DSM‐III‐R) *Review* Heterogeneity: diff countries, response rate, sample size	5
Baxter 2013 *Search:* 2009 *# incl. studies* 87 *Meta‐analysis:* yes	Further research on: Impact of conflict on mental health Aspects of wealth related to anxiety Cultural aspects (ex. psycho‐stressors) related to anxiety Further studies using consistent anxiety definition and methodologies in 1) developing and emerging countries; 2) populations exposed to conflict Interactions of factors associated with prevalence of anxiety	*Original studies* Limited measurement equivalence across cultures – results should be interpreted with caution Rural study results – should be interpreted with caution Study design differences *Review* NR	10
Mirza 2004 *Search:* March 2002 *# incl. studies:* 20 *Meta‐analysis:* no	Robust evidence (ex. conduct national, mental health epidemiology surveys) to develop mental health policy with strategic implementation plan for Pakistan More outcome studies, prevention and treatment trials needed	*Original studies* Most studies from Punjab and Sind Heterogeneity in study design and instruments – limited generalizability *Review* Publication and selection bias Small number of included studies	5
Vehling 2012 *Search: not rep.* *# incl. studies* 89 *Meta‐analysis:* yes	Representative studies	*Original studies* Estimate heterogeneity and study quality Limited generalizability *Review*	7
Baxter 2014 *Search:* 2009 *# incl. studies* 91 *Meta‐analysis:* yes		*Original studies* Limited or no data from Central Asia, Andean Latin America, Oceania, Central sub‐Saharan Africa, Central Europe, South‐east Asia Possibly biased population samples (ex. conflict region studies may have oversampled those exposed to conflict) *Review* NR	10
Haller 2014 *Search:* 2006 *# incl. studies:* 18 *Meta‐analysis:* no	Clarify subthreshold GAD vs. nonpathological anxiety – use impairment criterion for this Should treatment strategies used for threshold disorders be used for subthreshold cases?	*Original studies* Inadequate study response rates Heterogeneous definitions of subthreshold GAD *Review* Some studies missed Difficult to define search terms for subthreshold GAD Insufficient studies for subpopulations Different study quality	7
Steel 2014 *Search:* Jan 2014 *# incl. studies* 174 *Meta‐analysis:* yes		*Original studies* Some recall bias with 12‐month estimates Different study age structures contributing to different prev Higher prev with smaller sample sizes Different estimates with the use of different instruments Adaptation of surveys to culture and context & measurement equivalence issues *Review* Some studies may have been missed Untested search strategies Assessment equivalence across cultures Can only generalize findings to adults	5
**Addiction**			
Fatseas 2010 *Search:* Jan. 2009 *# incl. studies* 18 *Meta‐analysis:* no	Effectiveness of treatment for phobias in opiate‐dependent patients	*Original studies* Reliability and validity of diagnostic tools (ex. difficult to distinguish substance‐induced anxiety from independent disorders with pre‐DSM‐IV criteria) Heterogeneity in sample characteristics Different time frames for prev of anxiety *Review*	6
Fischer 2012 *Search:* Dec. 2011 *# incl. studies* 9 *Meta‐analysis:* yes	Longitudinal studies to assess reasons for using NMPOU in individuals with mental health problems	*Original studies* Heterogeneity: operationalization of anxiety and NMPOU Many screener or epidemiological instruments used (possible overestimation), instead of clinical diagnostic tools All North American studies – limited generalizability Small number of studies *Review* Between‐study heterogeneity	8
Goldner 2014 *Search:* April 2012 *# incl. studies* 11 *Meta‐analysis:* yes	Relationship between NMPOU and mental illness Retrospective and prospective studies to examine development of mental health problems and NMPOU in those receiving POAs Use standardized and comparable diagnostic instruments Link between chronic pain and mental illness Alternative treatments for and outcomes of patients with both mental health problems and NMPOU	*Original studies* Cross‐sectional data, thus temporality issues between NMPOU and mental illness Diff instruments used *Review* Publication bias High between‐study differences Heterogeneity: defining and measuring NMPOU psychiatric problems	8
Lorains 2011 *Search:* Sept. 2010 *# incl. studies* 11 *Meta‐analysis:* yes	Health care workers should: Assess for comorbidities Determine whether anxiety developed before gambling problem and should be treated first	*Original studies* Lifetime estimates may be confounded by age Diff tools (ex. SOGS – satisfactory psychometrics in populations surveys; discordance between NODS and DSM‐IV) Most general population prevalence surveys conducted in the United States and Canada, small sample sizes *Review* NR	5
Ho 2014 *Search:* 2012 *# incl. studies* 8 *Meta‐analysis:* yes	Genetic transmission of IA Patients with IA should be screened for anxiety and vice versa & integrated treatment recommended Further studies on moderators; other ethnic groups in Europe and North America; older adults Studies on interactions between IA and anxiety (etiology, illness trajectory, treatment outcomes) Consensus on definition of IA Prospective studies Link between anxiety and IA‐specific behaviors (ex. use of social media)	*Original* Heterogeneity: age of sample, different psychiatric questionnaires, mostly cross‐sectional studies, uncontrolled confounding (ex. environmental stress, parenting) Young patients mainly from Asian countries *Review* Small number of studies Unable to assess how estimates differ with use of self‐reported questionnaires vs. structured interviews	8
**Other mental and neurological disorders**			
Fajutrao 2009 *Search:* past 10 years *# incl. studies* 26 *Meta‐analysis:* no	Bipolar disorder in Europe	*Original studies* Anxiety assessment and reporting methods diff Retrospective and nonrepresentative samples *Review* Focus on electronic databases; language selection criteria	5
Amerio 2014 *Search:* Mar 2013 *# incl. studies:* 64 *Meta‐analysis:* no	Assess history of mood disorders in OCD patients Treatment research (ex. use of mood stabilizers) Studies on hereditary and biological markers, diagnostic validity of BD‐OCD comorbidity and its treatments	*Original studies* Differences in evaluation, diagnosis, reporting Mostly observational, retrospective studies, lack of control group, small sample size, sampling bias *Review* NR	5
Swets 2014 *Search:* Dec 2009 *# incl. studies* 43 *Meta‐analysis:* yes	Use random sampling Training needed to assess OCS Diagnostic standardization needed, careful patient selection Detailed assessment of OCD; use SCID OCD def. followed by Y‐BOCS administration Assess OCS in patients with psychosis Shift from descriptive to treatment studies	*Original studies* Different instruments and criteria used (ex. lower estimates with DSM‐III‐R than later versions; lower prev with DIGS) Sampling variability (different patient characteristics) Possible sampling bias, help‐seeking/patients selection can influence prev rates Limited data on: sub‐Sahara African countries, gender, ethnicity, use of meds (ex. antipsychotics) *Review* NR	5
Marrie 2015 *Search:* Nov. 2013 *# incl. studies* 118 *Meta‐analysis:* yes	Be consistent: compare psychometric properties of instruments and use same instrument to assess anxiety Standardize estimates to common (world) population	*Original* Differences in study design: different data sources, populations, definitions of psychiatric disorders Little info on age‐, sex‐, or ethnicity‐specific estimates *Review* NR	5
**Chronic physical diseases**			
*Cardiovascular disease*			
Janssen 2008 *Search:* 2007 *# incl. studies* 39 *Meta‐analysis:* no	Prospective research that considers view of patients, their families, their physician for symptom management	*Original studies* Different rates of symptom reporting with different proxies and depending on timing of interview Differences in: methods of reporting; definition of end‐of‐life (ex. different estimates in last week vs. last year of life); patient characteristics; definition and measurement of symptoms *Review* NR	5
Solano 2006 *Search:* June 2004 *# incl. studies* 64 *Meta‐analysis:* no		*Original studies* Heterogeneity in definition of symptoms (different criteria), methods to detect cases of symptoms (different questionnaires and screening methods used), study design, sampling, study setting, methods of data collection	5
Tully 2013 *Search:* May 2011 *# incl. studies* 12 *Meta‐analysis:* yes	Further GAD research in CHD Specific anxiety disorders rather than trait/state anxiety “Any anxiety” not clinically informative in cardiac settings	*Original studies* Heterogeneity: diagnostic criteria for GAD, gender ratio, patient age *Review* Low rate of publications on GAD	6
Clarke 2009 *Search:* May 2003 *# incl. studies* 159 *Meta‐analysis:* no	Effectiveness of interventions Large prospective studies Anxiety assessed in parallel with chronic conditions	*Original studies* Different rating tools & diagnostic criteria; low power *Review* Heterogeneity	7
Webster 2012 *Search:* Nov. 2010 *# incl. studies* 12 *Meta‐analysis:* no	Theory‐driven research to examine link between patients’ perceptions (ex. chest pain) and mental health Does providing explanations for patients’ chest pain reduce their anxiety? Longitudinal design to assess mental health trajectory in NCCP Use reliable and valid measures for mental disorders with recommended cut‐offs	*Original studies* Different caseness cut‐offs Risk factor research used cross‐sectional designs Few studies on correlates of poor mental health in NCCP *Review* Possible publication bias Large heterogeneity in study settings	5
Campbell Burton 2013 *Search:* March 2011 *# incl. studies* 44 *Meta‐analysis:* yes	Mood assessment tools appropriate for stroke patients Guidance on best time to screen for anxiety What is the impact of anxiety and its economic burden in the context of stroke?	*Original studies* Different cut‐off scores used Most studies cross‐sectional, so difficult to determine whether pre‐stroke anxiety is linked to post‐stroke anxiety Few studies differentiated btw. “first‐ever” and “current anxiety” Some scales were not validated in stroke populations *Review* Potential publication bias & heterogeneity Some studies not included in review	10
*Cancer*			
Clarke 2009 – previously described			
Solano 2006 – previously described			
Yang 2013 *Search:* Sep. 2012 *# incl. studies* 17 *Meta‐analysis:* yes	Use control groups with diseases other than cancer	*Original studies* Anxiety assessed using different instruments Studies were cross‐sectional so cannot determine temporality between anxiety and cancer development *Review* Few studies & lacking international literature Potential publication bias	9
Vehling 2012 – previously described			
Lim 2011 *Search:* 2010 *# incl. studies* 10 *Meta‐analysis:* no	Studies in different settings assessing effect of cancer treatment on anxiety Interventions for anxiety in women with breast cancer Ways to decrease state anxiety and help women cope with chemotherapy, despite their level of trait anxiety	*Original studies* Small sample sizes *Review* Difference in treatment, tools & timing of measurement	6
Arden‐Close 2008 *Search:* May 2007 *# incl. studies* 18 *Meta‐analysis:* no	Longitudinal studies and RCTs needed to clarify directionality between immunity and mental illness Prospective research needed to test trajectories of change in mental illness following cancer diagnosis and treatment Interventions targeting distress (ex. coping) Attention to sample size and validation of questionnaires Theory‐driven research needed Authors should state limitations/directions for future research	*Original studies* Certain correlates of mental illness tested in too few studies Lack of validation of assessment tools Small sample sizes Residual confounding Limited generalisability (US) *Review* Published studies	6
Mitchell 2013 *Search:* March 2013 *# incl. studies* 43 *Meta‐analysis:* yes	Link between health‐related quality of life and anxiety Studies on anxiety in palliative settings or in patients with advanced cancer More reliable estimates by use of interview methods	*Original studies* Differences in: quality of matching with healthy controls, study quality, study design, case ascertainment Possible uncontrolled factors Heterogeneity in healthy controls (review authors had limited info on recruitment of healthy controls in studies) *Review* – NR	11
*Respiratory disease*			
Janssen 2008 – previously described			
Solano 2006 – previously described			
Davydow 2008 *Search:* April 2007 *# incl. studies* 10 *Meta‐analysis:* no	Risk factors for psychopathology More rigorous assessment of psychopathology Anxiety in ICU as risk factor for post‐ALI/ARDS psychopathology To what extent are risk factors for ALI/ARDS related to development of mental illness in those without ALI/ARDS	*Original studies* Mostly psychiatric questionnaires used with diff. sensitivities, ex. screening instruments or measures of symptom severity (not necessarily validated for ARDS survivors) Small sample sizes *Review* Small number of studies	5
*Diabetes*			
Smith 2013 *Search:* July 2012 *# incl. studies* 12 *Meta‐analysis:* yes	Individual anxiety disorders associated with diabetes Relevant confounders should be included Studies on diabetes and anxiety using accurate measurements Prospective studies to clarify directionality between anxiety and diabetes	*Original studies* Different time frames resulting in different likelihood of capturing symptoms Measurement differences Cross‐sectional data Temporality between diabetes and anxiety *Review* Publication bias, language biases	10
Grigsby 2002 *Search:* 2001 *# incl. studies* 18 *Meta‐analysis:* yes	Longitudinal studies to identify behavioral and physiological mechanisms related to anxiety in diabetes More community‐based studies to estimate anxiety prev in diabetes Assess potential moderators Studies on causal mechanisms	*Original studies* Small sample sizes Lacking data on race/ethnicity influence on anxiety prev Differences in scales used to measure anxiety and in aggregation/reporting of results (ex. assessment of 1 anxiety disorder vs. aggregate of several anxiety disorders) Lack of data on prev of anxiety by diabetes type *Review* Small number of studies Few studies included nondiabetic comparison group	6
Clarke 2009 – previously described			
**Other chronic physical diseases**			
Dokras 2012 *Search:* April 2011 *# incl. studies* 9 *Meta‐analysis:* yes	Effect of clinical or biochemical factors in relation to hyperandrogenism and anxiety in PCOS Link between PCOS‐specific characteristics and anxiety Larger sample sizes Longitudinal studies for insight into etiology and trajectory of anxiety in PCOS	*Original studies* Few studies on prev on anxiety in PCOS using validated anxiety screening tools Mostly cross‐sectional studies *Review* Small sample sizes, possible publication bias	5
Smith 2014 *Search:* January 2013 *# incl. studies* 14 *Meta‐analysis:* yes	Degree of BJHS related to mental illness Biological link between BJHS and anxiety (ex. abnormal reactive autonomic nervous system) Influence of nonpharmacologic treatment on alleviating anxiety in those with BJHS Anxiety in BJHS in other cultures	*Original studies* Limited generalizability (mainly Mediterranean adult populations), mostly cross‐sectional designs Possible cross‐cultural differences in expression of anxiety *Review*	7
Andersen 2014 *Search:* Sept. 2012 *# incl. studies* 24 *Meta‐analysis:* no		*Original studies* Different recruitment methods, study inclusion criteria Most study patients were women, thus, possible overestimation of significance of results Different measurement methods: questionnaires, clinical evaluations, structured interviews (some methods not validated for pain patients) *Review* Search strategy	6
Dawson 2014 *Search:* Feb 2012 *# incl. studies* 16 *Meta‐analysis:* no	Does anxiety come before onset of AMD? Link between length of time since AMD diagnosis and AMD treatments in relation to patient's mental health Include control group to compare prev of anxiety between AMD and non‐AMD populations Use tools with clear cut‐off for clinical anxiety	*Original studies* Is anxiety different in different forms of AMD? Different definition and measurement of anxiety Comparison group may not be representative *Review* Small number of studies	5
**Other chronic physical diseases in end‐stage**			
Mitchell 2011 *Search:* Nov. 2010 *# incl. studies* 94 *Meta‐analysis:* yes		*Original studies* No consensus about optimum psychiatric diagnostic approach in cancer settings Studies of variable quality, mostly cross‐sectional designs, some used convenience sampling, different anxiety measurement methods Could not determine correlates of anxiety Few studies with defined period of prevalence *Review* Possible publication bias	8
Janssen 2008 – previously described			
Murtagh 2007 *Search:* April 2005 *# incl. studies* 60 *Meta‐analysis:* No	Studies on incidence and prevalence of symptoms in ESRD, their causes, and interventions Population‐based, longitudinal studies More information on generalizability of available studies How do symptoms vary between those managed without dialysis and those withdrawing from dialysis? Symptom burden in ESRD Symptoms experienced at end of life Identify what is common and different between those dying from ESRD and other palliative populations	*Original studies* Heterogeneity: symptom definition, who defines a symptom (reporting), different periods over which prevalence is measured, different tools used No population‐based studies *Review* Search strategy	6
Solano 2006 – previously described			
**Trauma**			
Mckechnie 2014 *Search:* June 2013 *# incl. studies* 13 *Meta‐analysis:* no	Prospective studies assessing long‐term levels of anxiety in post‐traumatic amputees, and whether rehab programmes are successful and mental health issues continue after the programme ends	*Original* No info on how prev changes with time since amputation (anxiety assessed at fixed time point) Different scoring systems in different populations at various follow‐up times Selected specialist samples not representative of all traumatic amputees Sampling – possible selection bias Attrition during follow‐up *Review* Some studies may have been missed	8
Chen 2010 *Search:* Dec. 2008 *# incl. studies* 37 studies *Meta‐analysis:* yes	Interplay between stressful life events, vulnerability genes, and development of psychiatric disorders (gene‐environment interactions)	*Original studies* Self‐report (recall bias), abuse underreport Anxiety affected by unmeasured forms of abuse? *Review*	8
Fazel 2005 *Search:* Dec. 2002 *# incl. studies* 20 *Meta‐analysis:* yes		*Original studies* Measurement equivalence issues: differences in sampling methods, diagnostic instruments Insufficient data on refugees in developing countries, asylum seekers, people internally displaced in their own countries Updated info on recently displaced refugees *Review* NR	5
**Vulnerable population subgroups**			
*Older people and their caregivers*			
Bryant 2008 *Search:* 2007 *# incl. studies* 49 *Meta‐analysis:* no	Hypothesis‐driven research with late‐life anxiety as primary focus Longitudinal designs Studies on anxiety in old age Prevention and early treatment should target old people in poor health and who are at risk for anxiety	*Original studies* Differences in definition and measurement of anxiety Measurement equivalence issues in elderly – is anxiety experienced differently in elderly? (case definition) Difficult to disentangle physical symptoms & anxiety in elderly Possible selection bias Older people may underreport anxiety Mostly cross‐sectional studies *Review*	5
Volkert 2013 *Search date:* Dec. 2011 *# incl. studies* 25 *Meta‐analysis:* yes	Studies on anxiety in elderly using improved methodology and accounting for changes in old age (adapted instruments)	Differences in instruments and diagnostic criteria Difficult to disentangle anxiety from physical diseases, somatoform disorders, and depression in elderly Instruments not designed for elderly – what constitutes anxiety in elderly? Heterogeneity: studies of different geographic and cultural regions and using different case definitions and case identification methods Difficult to recruit elderly for studies *Review* Studies in English and German – limited generalizability No missing data analysis	8
Monastero 2009 *Search:* Aug. 2008 *# incl. studies* 27 *Meta‐analysis:* no	Health care worker to distinguish primary behavioral changes from cognitive impairment Large, cohort studies using standardized instruments to assess NPS as prognostic factors in MCI Optimum ways to assess NPS in those with MCI Genetic and biological markers linking NPS to MCI and dementia	*Original studies* Possible selection bias Differences in age and sex distributions within studies Differences in instruments used/methods of reporting symptoms *Review* NR	5
Yates 2013 *Search:* Nov. 2012 *# incl. studies* 18 *Meta‐analysis:* yes	Anxiety and depression should both be considered Classification systems for MCI should consider anxiety Clarify directionality between anxiety and MCI	*Original studies* Heterogeneity: sampling differences, small samples (may not be representative), different ways of assessing mood/NPS Lacking info on link between anxiety and MCI subtypes *Review* Possible publication bias, English articles	5
Cooper 2007 *Search:*2005 *# incl. studies* 33 *Meta‐analysis:* no	Cohort studies Research on coping in relation to anxiety (this could be intervention target)	*Original studies* Lack of info on determinants of anxiety caseness in caregivers *Review* NR	5
*Pregnant women*			
Russell 2013 *Search:* August 2012 *# incl. studies* 17 *Meta‐analysis:* yes	Prospective studies examining OCD during pregnancy and postpartum period Incidence studies needed Course of OCD across reproductive events Influence of biological determinants on OCD exacerbation throughout reproductive period	*Original studies* Small samples Difficult to match control studies on various factors Possible overestimation of OCD prev in some control studies OCD evaluated at different pregnancy time points, making comparisons difficult *Review* Published studies	8
Molyneaux 2014 *Search:* Jan 2013 *# incl. studies* 62 *Meta‐analysis:* insufficient studies for meta‐a for anxiety	Validation of anxiety scales for specific populations needed, ex. women in early pregnancy	*Original* Heterogeneity: different screening measures and cut‐offs *Review* English language papers only Published studies eligible Few studies carried out in low and middle‐income countries	6
Sawyer 2010 *Search:* January 2009 *# incl. studies* 35 *Meta‐analysis:* yes	Longitudinal studies to determine anxiety prev at different time points during and after pregnancy Develop cross‐cultural measures of mental health	*Original studies* Small number of studies Measurement issues, timing of mental health assessment varied (thus, anxiety trajectory over time is unclear) Few studies on antenatal mental health and associated risk factors in African women Insufficient info on how maternal psychological problems impact children *Review* – NR	6
*LGB and self‐harm patients*			
King 2008 *Search:* 2005 *# incl. studies* 25 *Meta‐analysis:* yes	Prospective studies to determine risk factors of mental disorders Refine definition of sexual orientation	*Original studies* Difficult to recruit and define LGB group Study design heterogeneity Heterogeneity in definitions of exposure and outcome *Review* Heterogeneity: study designs and LGB definition Small number of studies included	7
Hawton 2013 *Search:* Nov. 2011 *# incl. studies* 50 *Meta‐analysis:* yes	Studies on mental disorders in those who repeat self‐harm	*Original studies* Measurement equivalence issues Heterogeneity: methods used to recruit participants, different diagnostic measures used, differences in study participant gender ratios cross‐sectional studies *Review* English language studies	6

a prev, prevalence; anx, anxiety; NR, not reported; QA, quality assessment based on AMSTAR criteria.
